# Patterns of Promotional Content by Dermatology Influencers on TikTok

**DOI:** 10.2196/34935

**Published:** 2022-03-30

**Authors:** Varun K Ranpariya, Ramie Fathy, Brian Chu, Sonia Wang, Jules B Lipoff

**Affiliations:** 1 Rutgers Robert Wood Johnson Medical School Rutgers University Piscataway, NJ United States; 2 Perelman School of Medicine University of Pennsylvania Philadelphia, PA United States; 3 Department of Dermatology Perelman School of Medicine University of Pennsylvania Philadelphia, PA United States; 4 Leonard Davis Institute of Health Economics University of Pennsylvania Philadelphia, PA United States

**Keywords:** social media, TikTok, Instagram, promotion, conflicts of interest, influencer, dermatology, dermatologist

## Research Letter

TikTok, a social media platform for sharing short videos, has become a source of dermatologic information for the general public [1,2]. Compared to other platforms, TikTok has high engagement rates (ratio of likes and comments to followers)—approximately 5 times those of Instagram [1]. The platform is rife with promotional content [1,2], potentially influencing public behavior and consumption, such as boosting CeraVe’s sales in early 2021 [3]. Here, we sought to characterize promotional content among accounts with the most popular dermatology-related TikTok videos.

We analyzed 14 hashtags to identify the top dermatology TikTok videos for analysis of promotional content. Our hashtags were based on precedent social media studies and included the top 5 dermatology-related diagnoses and the top 5 dermatology procedures [4]. We also added 4 hashtags anecdotally found to be popular on TikTok (Table 1). The top 100 posts for each hashtag were queried on February 26, 2021, totaling 1400 posts. Based on the precedent for identifying Instagram influencers, we employed two criteria to define influencer status [4]. The first criteria required accounts to have ≥500,000 followers; the second required being featured in the top 100 posts across all hashtags ≥3 times. Promotional content was defined per the Federal Trade Commission: any disclosures (hashtags, text, or video content indicating advertisement, ambassadors, discounts, or tags) in the influencers’ 9 most recent posts or biography [5]. Similarly, personal promotion was defined as disclosures promoting the influencers’ own products or services.

**Table 1:** Hashtags queried in the study and the change in the total number of views for each hashtag over 3 months (February 14 to May 14, 2021).

Hashtag	Views, n		Difference, n
	February 14, 2021	May 14, 2021	
#skincare	31,200,000,000	41,600,000,000	10,400,000,000
#dermatologist	1,600,000,000	2,700,000,000	1,100,000,000
#dermatology	457,500,000	640,800,000	183,300,000
#skincareroutine	7,000,000,000	8,900,000,000	1,900,000,000
#acne	6,800,000,000	9,500,000,000	2,700,000,000
#eczema	77,300,000	132,900,000	55,600,000
#psoriasis	85,900,000	137,900,000	52,000,000
#hairloss	496,800,000	736,100,000	239,300,000
#alopecia	1,100,000,000	1,500,000,000	400,000,000
#botox	669,000,000	1,100,000,000	431,000,000
#juvederm	31,700,000	37,100,000	5,400,000
#microneedling	133,000,000	179,700,000	46,700,000
#laserhairremoval	139,500,000	252,700,000	113,200,000
#dermalfillers	18,600,000	32,300,000	13,700,000
Total	49,809,300,000	67,449,500,000	17,640,200,000

From February 14 to May 14, 2021, TikTok videos with hashtags of interest accumulated 17.6 billion views (Table 1). Of the 1400 posts recorded, there were 1337 unique posts from 738 unique accounts. After excluding non-English–language posts and accounts with posts unrelated to dermatology, 112 accounts remained with ≥500,000 followers and 77 accounts featured ≥3 times in the top 100, totaling 162 accounts meeting one or both influencer criteria (Table 2). Of this total, 14 (8.6%) were dermatologists, with 8 out of 14 being board-certified. Over one-third (57/162, 35.2%) of these influencers had promotional content on their account, and 32.1% (52/162) had personal promotional content. Promotional status was undetermined in 15.4% (25/162) of accounts (non-English).

About 35% of dermatology influencers featured promotional content on TikTok, which raises concerns about conflicts of interest. Although dermatologists represent a fraction of influencers, a majority (8/14, 57.1%) featured promotional content. Noncredentialled, dermatology-related accounts had the highest rate of promotional content (22/28, 78.6%), which included skincare brand partnerships, product links, and personalized discount codes. Disclosures, which can be indicated using *#ad* in the video descriptions or explicitly mentioning conflicts in the videos, should be stated in user biographies, especially when providing product links with affiliate marketing incentives. Additionally, clearly stating a lack of conflict when recommending or reviewing products could reduce perceptions of conflict.

Given the prevalence of nondermatology and nonmedical influencers creating dermatology content, leveraging TikTok to counter misinformation may be essential to ensure patients and health consumers are provided accurate information. While new avenues to share educational content are important, the negative influence of promotional content remains a concern.

**Table 2:** Characterization of TikTok influencer types and promotional content patterns.

Characteristic			Accounts, n (% of all influencers)	Accounts, n (% within subcategory)			
				Promotional	Personal promotion	None	Unknown (non-English)
**Influencer category**							
	All		162 (100)	57 (35.2)	52 (32.1)	28 (17.3)	25 (15.4)
	≥500,000 followers		112 (69.1)	45 (40.2)	31 (27.7)	20 (17.9)	16 (14.3)
	≥3 times in the top 100		77 (60.2)	28 (36.4)	27 (35.1)	10 (13.0)	12 (15.6)
**Account type**							
	Personal		66 (40.7)	16 (24.2)	23 (34.9)	22 (33.3)	5 (7.6)
	**Physician^a^**		5 (3.1)	1 (20.0)	2 (40.0)	0 (0)	2 (40.0)
		Board-certified^b^	1 (0.6)	1 (100)	0 (0)	0 (0)	0 (0)
		Not board-certified^b^	1 (0.6)	0 (0)	1 (100)	0 (0)	0 (0)
		International	3 (1.9)	0 (0)	1 (33.3)	0 (0)	2 (66.7)
		Resident	0 (0)	0 (0)	0 (0)	0 (0)	0 (0)
	**Dermatologist**		14 (8.6)	8 (57.1)	4 (28.6)	1 (7.1)	1 (7.1)
		Board-certified^b^	8 (4.9)	5 (62.5)	2 (25.0)	1 (12.5)	0 (0)
		Not board-certified^b^	0 (0)	0 (0)	0 (0)	0 (0)	0 (0)
		International	1 (0.6)	0 (0)	0 (0)	0 (0)	1 (100)
		Resident	5 (3.1)	3 (60.0)	2 (40.0)	0 (0)	0 (0)
** **	**Plastic surgeon**		4 (2.5)	1 (25.0)	3 (75.0)	0 (0)	0 (0)
		Board-certified^b^	3 (1.9)	1 (33.3)	2 (66.7)	0 (0)	0 (0)
		Not board-certified^b^	0 (0)	0 (0)	0 (0)	0 (0)	0 (0)
		International	1 (0.6)	0 (0)	1 (100)	0 (0)	0 (0)
		Resident	0 (0)	0 (0)	0 (0)	0 (0)	0 (0)
	Nurse, nurse practitioner, physician assistant, or advanced practitioner		7 (4.3)	2 (28.6)	3 (42.9)	2 (28.6)	0 (0)
	Esthetician		5 (3.1)	2 (40.0)	2 (40.0)	0 (0)	1 (20.0)
	Dermatology or skincare informational company account (no individual user identified)		5 (3.1)	1 (20.0)	2 (40.0)	1 (20.0)	1 (20.0)
	Dermatology- or skincare-focused account with no credentials		28 (17.3)	22 (78.6)	6 (21.4)	0 (0)	0 (0)
	Other		5 (3.1)	0 (0)	3 (60.0)	2 (40.0)	0 (0)
	Unknown (non-English language)		23 (14.2)	4 (17.4)	4 (17.4)	0 (0)	15 (65.2)
**Location**							
	United States		100 (61.7)	43 (43.0)	43 (43.0)	14 (14.0)	0 (0)
	International		42 (25.9)	10 (23.8)	6 (14.3)	6 (14.3)	20 (47.6)
	Unknown		20 (12.4)	4 (20.0)	3 (15.0)	8 (40.0)	5 (25.0)

^a^Physicians not including dermatologists or plastic surgeons.

^b^Per the American Board of Medical Specialties [6].
